# Local YB-1, Epo, and EpoR concentrations in fractured bones: results from a porcine model of multiple trauma

**DOI:** 10.1186/s40001-023-00996-w

**Published:** 2023-01-13

**Authors:** Felix Marius Bläsius, Johannes Greven, Weijun Guo, Eftychios Bolierakis, Zhizhen He, Cavan Lübke, Tim-Philipp Simon, Frank Hildebrand, Klemens Horst

**Affiliations:** 1 Deptartment of Orthopaedics, Trauma and Reconstructive Surgery, University Hospital, RWTH University, Pauwelsstraße 30, 52074 Aachen, Germany; 2 Insitute of Pharmacology and Toxicology, University Hospital, RWTH University, Aachen, Germany; 3 Department of Intensive Care and Intermediate Care, University Hospital, RWTH University, Aachen, Germany

**Keywords:** YB-1, Erythropoietin, Epo, Erythropoietin receptor, Multiple trauma, Traumatic hemorrhage, Shock, Porcine model

## Abstract

Little is known about the impact of multiple trauma (MT)-related systemic hypoxia on osseous protein concentration of the hypoxia transcriptome. To shed light on this issue, we investigated erythropoietin (Epo), erythropoietin receptor (EpoR), and Y-box binding protein 1 (YB-1) concentrations in the fracture zone in a porcine MT + traumatic hemorrhage (TH) model. Sixteen male domestic pigs were randomized into two groups: an MT + TH group and a sham group. A tibia fracture, lung contusion, and TH were induced in the MT + TH group. The total observation period was 72 h. YB-1 concentrations in bone marrow (BM) were significantly lower in the fracture zone of the MT + TH animals than in the sham animals. Significant downregulation of BM-localized EpoR concentration in both unfractured and fractured bones was observed in the MT + TH animals relative to the sham animals. In BM, Epo concentrations were higher in the fracture zone of the MT + TH animals compared with that in the sham animals. Significantly higher Epo concentrations were detected in the BM of fractured bone compared to that in cortical bone. Our results provide the first evidence that MT + TH alters hypoxia-related protein concentrations. The impacts of both the fracture and concomitant injuries on protein concentrations need to be studied in more detail to shed light on the hypoxia transcriptome in fractured and healthy bones after MT + TH.

## Introduction

Fractures are one of the most common injuries after multiple trauma (MT) [1]. Timely and uneventful healing are essential for rapid rehabilitation in MT cases [2]. Both local inflammation and hypoxia at the fracture site are key factors in the onset of uneventful bone healing [3–5]. In this context, previous data from our and other groups suggested that MT and associated specific injury patterns (e.g., chest trauma and traumatic hemorrhage [TH]) have a pronounced impact on the local inflammatory response at the fracture site [6, 7], thereby potentially affecting bone healing.

In the early fracture healing process, local hypoxia promotes angiogenesis, chemotaxis, and osteogenesis at the fracture site [3–5]. In cases of accompanying chest trauma and TH, MT has a significant impact on the systemic oxygenation. In this regard, animal experiments have provided evidence that systemic hypoxia influences fracture healing, presumably due to disturbance of the local inflammatory response, osteoclast recruitment, differentiation, and ultimately, callus formation [8]. Despite the impact of systemic hypoxia on the oxygen supply, data on the influence of MT + TH on hypoxia-dependent gene regulation at the fracture site (commonly referred to as the hypoxia transcriptome) and related protein concentrations are not available. Among the systems of the hypoxia transcriptome, erythropoietin (Epo) concentration, erythropoietin receptor (EpoR), and cold shock protein Y-box binding protein 1 (YB-1) concentrations respond rapidly and sensitively to hypoxia [9, 10]. In this context, YB-1 expression can be dramatically upregulated within 8 h of hypoxia and deactivated just as rapidly [11]. Recently, it was shown by Rauen et al. that depletion of cellular YB-1 leads to transcriptional upregulation of the EPO-hypoxia-responsive element (Epo-HRE). In this context, increased YB-1 expression under hypoxia acted as an antagonist to hypoxia-inducible factor (HIF) family-induced remote regulation of Epo-HRE [9]. In addition, known target genes of the hypoxia transcriptome such as vascular endothelial growth factor (VEGF) are inhibited by YB-1 [12]. Therefore, these parameters seem to be particularly suitable for assessing the changes of the hypoxia-related transcriptome at fracture sites after MT + TH. Information about trauma-induced changes in protein concentrations in the early post-traumatic phase are of major importance, as such changes and cell–cell interactions likely influence the initiation of the bone healing process and thus physiological fracture healing.

In this study, we investigated Y-box binding protein 1, Epo, and EpoR concentrations in a well-established and clinically relevant porcine model of MT + TH at the fracture site. We also examined whether MT affected the YB-1 signaling axis in uninjured bone. Due to the different regenerative potential of cortical bone (CB) and bone marrow (BM) [13], we focused specifically on these two osseous compartments.

## Materials and methods

### Animal care

The animal model used has been described earlier [14] and was modified in accordance with the experimental purpose of the present study [15]. This study was performed in accordance with the 3R criteria of Russell and Burch. The results presented herein form part of a larger study authorized by the governmental animal care and use office (Authorization no. AZ 81-02.04.2017.A412; Landesamt für Natur, Umwelt und Verbraucherschutz Nordrhein-Westfalen, Recklinghausen, Germany) that was performed at the Institute for Laboratory Animal Sciences, University Hospital, RWTH University, Aachen, Germany. Therefore, the experiments were performed in accordance with European (EU Directive 2010/63/EU) and national animal welfare law (TierSchG, Federal Republic of Germany). Details on the experimental setup can be found in our earlier studies [15, 16].

The study included 16 male domestic pigs (German Landrace, *sus scrofa*, 35 ± 5 kg body weight). All the animals underwent a clinical examination by a veterinarian prior to inclusion in the study and were housed in a ventilated facility to allow acclimatization to their surroundings for a minimum of 7 days before the experiments started. Treatment, housing, and husbandry conditions conformed to European Union Guidelines (Directive 2010/63/EU on the protection of animals used for scientific purposes). MT and TH were induced in eight pigs, and eight pigs served as instrumented but noninjured controls (sham group).

### General instrumentation and anesthesia

After a 12-h fasting period, during which the animals had access to water ad libitum, the animals were premedicated. For premedication, the animals received an intramuscular injection of azaperon (Stresnil™; Elanco Deutschland GmbH, Bad Homburg v.d.H., Germany). Anesthesia was induced by an intravenous injection of remimazolam (PAION AG, Aachen, Germany), followed by orotracheal intubation (Hi-Lo Lanz™; Medtronic GmbH, Germany). Mechanical ventilation in the volume-controlled mode (Evita 4; Draeger AG & Co. KGaA, Luebeck, Germany) was applied (6–8 ml/kg), with an inspiratory oxygen fraction (FiO_2_) of 0.5 and a positive end-expiratory pressure of 8 mmHg (plateau pressure: < 30 mmHg). The tidal volume was adjusted to a target partial pressure of carbon dioxide value of 35–45 mmHg as indicated for long-term ventilation of severely injured patients [17]. Vital parameters were monitored by electrocardiographic recordings and pulse oximetry as previously described [14].

General anesthesia was maintained with isoflurane, fentanyl, and remimazolam. Fluids were administered continuously by intravenous infusion of crystalloids as previously described [15].

A central venous 8.5 Fr. four-lumen catheter (Arrow-Howes™; Teleflex Medical GmbH, Fellbach, Germany) was placed in the external jugular vein for administration of fluids, anesthesia, and continuous monitoring of central venous pressure. A two-lumen 12 Fr. large-bore hemodialysis catheter (Teleflex Medical GmbH, Fellbach, Germany) was placed in the left femoral vein for hemorrhage induction and blood sampling. An arterial line using pulse contour cardiac output analysis and a 5 Fr. pulse contour cardiac output catheter ([PiCCO] GETINGE; Maquet GmbH, Rastatt, Germany) was placed in the right femoral artery for continuous monitoring of hemodynamic parameters (e.g., mean arterial pressure and cardiac output). All intravascular pressure measurements were referenced to the mid-chest level. Finally, a suprapubic 14 Fr urine catheter (Roeseler, Germany) was placed via a mini-laparotomy.

### Sham group

Except for trauma and hemorrhage induction, the instrumentation, anesthesia, and intensive care management in the sham group were identical to the steps in the MT + TH group. The MT + TH group included a midline mini-laparotomy, which was performed for liver tissue sampling before trauma induction (liver sampling not relevant to this study).

### Induction of MT and hemorrhage

**Fig. 1:**
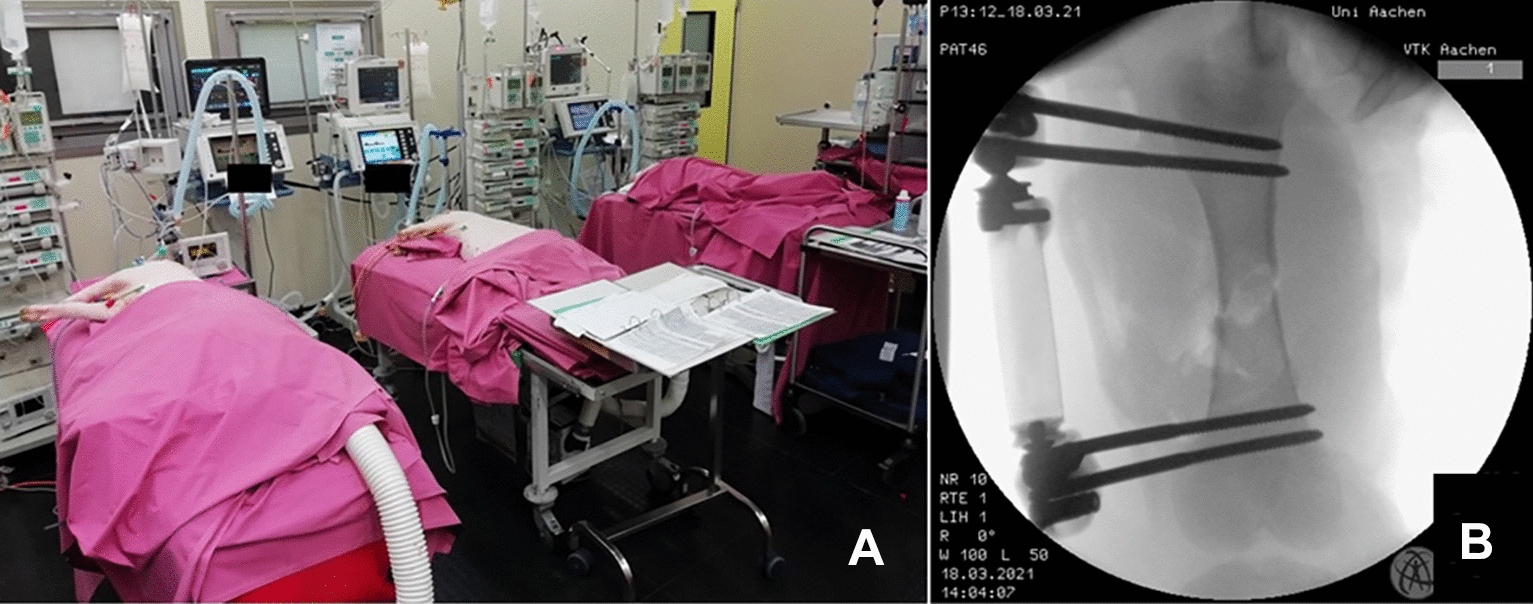
**A** Intensive care experimental setup. **B** C-arm X-ray confirming the diaphyseal tibia fracture

MT + TH was induced as previously described by Horst et al. [14] In brief, trauma was induced under FiO_2_ of 0.21, with fluid administration reduced to 0.1 ml/hour and hypothermia onset left untreated. A diaphyseal tibia fracture was made using a bolt gun (Schlachtapparat Blitz von Jopp Cal. 9 × 17; RUAG Ammotec GmbH, Fürth, Germany) (Fig. 1B). The fracture was confirmed by a clinical examination. Blunt thoracic trauma on the left side of the chest was induced as previously described using the above-mentioned bolt gun device [14]. Abdominal trauma consisted of a mini-laparotomy and exploration of the left upper liver lobe. Meanwhile, TH was induced by pressure-controlled withdrawing venous blood from the dialysis catheter until mean arterial pressure of 40 ± 5 mmHg was reached. At most, 45% of the total blood volume was withdrawn. The trauma phase lasted for 90 min.

The resuscitation phase (60 min) then started and included FiO_2_ adjustment to 0.5, blood re-infusion, continuous fluid administration, and rewarming (target temperature: 38.5–39.5 °C). Afterward, single-shot antibiotic prophylaxis with 2 g of ceftriaxone was administered, and surgical fixation of the fracture via external fixation (Orthofix^®^; Medical Inc., TX, USA) was performed during the surgical phase (60 min).

### Postoperative care

After stabilization, the intensive care period started. During mechanical ventilation, the animal’s position was changed every 6–8 h. A warm air blower was used to maintain the temperature of the animals within the physiological range. The animals received antibiotics (2 g of ceftriaxone) every 24 h and were kept anaesthetized using appropriate analgesia in a ventilated state until the end of the observation period (72 h). The intensive care experimental setup is shown in Fig. 1A.

### Bone sampling

BM and CB samples were taken after euthanasia (72 h after trauma). The samples were snap frozen in liquid nitrogen and stored at − 0 °C. For further analysis, tissue samples were homogenized using a T10 basic ULTRA-TURRAX^®^ (IKA-Werke GmbH & Co. KG, Staufen, Germany) with RIPA lysis and extraction buffer (Sigma-Aldrich, St. Louis, USA) and incubated for 10 min at 37 °C, with slight shaking. The protein was extracted in a ratio of 1 mg/10 µl RIPA buffer. After incubation, the solution was centrifuged at 2.500×*g* for 5 min to pellet the debris. Finally, the supernatant was either used for the analyses or snap frozen in liquid nitrogen and stored at − 80 °C until use.

### Western blot

**Fig. 2:**
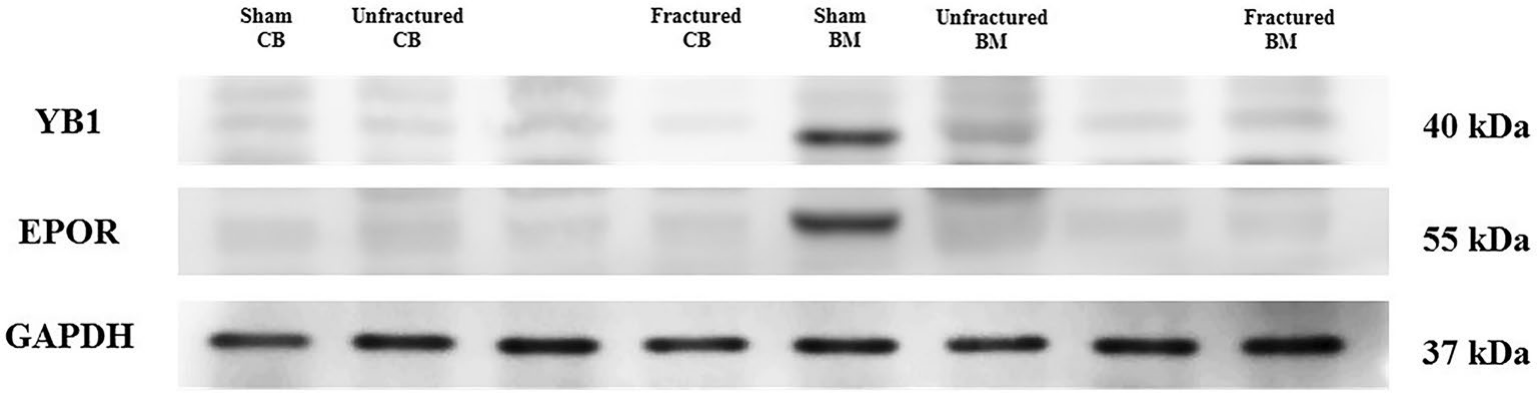
Exemplary Western blot bands used for chemiluminescence detection. Band intensities were analyzed and compared using ImageJ software (ImageJ, US. National Institutes of Health, Bethesda, Maryland, USA) and SPSS Version 27.0 (IBM Corp., Armonk, NY, USA)

The samples were grinded completely and dissolved in RIPA buffer and centrifuged for 20 min at 12,000 rpm at 4 °C in a microcentrifuge. The supernatants were then collected for further analyses. The protein concentration of the lysate was determined using a bicinchoninic acid protein assay kit (Thermo Scientific, USA). Equal amounts (30 μg) of protein per sample were separated by 10% sodium dodecyl sulfate–polyacrylamide gel electrophoresis and transferred to a polyvinylidene difluoride membrane. Membranes were blocked with 5% bovine serum albumin (Sigma-Aldrich) in Tris-buffered saline 50 mM Tris–HCl pH 7.6, 150 mM NaCl) containing 0.1% Tween-20 (Sigma-Aldrich) for 2 h. For antigen detection, the following antibodies were used: rabbit anti-GAPDH antibody (1:1000, LifeSpan Bioscience, LS-C108028), rabbit anti-YB-1 antibody (1:1500, LifeSpan Bioscience, LS-B5607), and rabbit anti-EpoR antibody (1:1000, LifeSpan Bioscience, LS-C761094). The blots were washed three times with Tris-buffered saline plus 0.1% Tween-20 and incubated for 1 h at room temperature (22 °C) with goat anti-rabbit immunoglobulin G (IgG) secondary antibody (1:5000, Abcam, ab6721). Immunoreactive bands were developed using an enhanced chemiluminescence detection system (ECL Plus; Thermo Scientific). Band density was quantified using ImageJ software (ImageJ, US National Institutes of Health, Bethesda, Maryland, USA). The protein concentration is depicted as the target/reference ratio of density values (Fig. 2).

### Enzyme-linked immunosorbent assay (ELISA)

An ELISA (MBS702779; MyBioSource Inc., CA, USA) was performed according to the manufacturer’s instructions. The Sunrise™ System (Tecan Trading AG, Maennedorf, Switzerland) was used for the ELISA readout.

### Statistical analysis, power and sample size estimation

Due to the fact that no information on mean values and standard deviations for Epo, EpoR, and YB-1 concentrations in bone after major trauma were available so far. An a priori power analysis was performed using lactate concentrations (shock model) as previously described. A group size of 8 animals yielded a power of 80% with a type I error of 0.05. An independent sample *t*-test was used to test the mean differences between the sham and trauma groups (two-tailed). A dependent *t*-test was used to test the mean differences between related samples (fracture zone vs. unfractured bone, two-tailed). In case of detection of unequal variances by applying Levene's test, nonequal variances were assumed and the corresponding test statistic was reported. The level of significance was set at α = 0.05. All data are presented as mean and standard deviation. The statistical analysis was performed using SPSS version 27.0 (IBM Corp., Armonk, NY, USA).

## Results

### Relative YB-1 concentrations

**Fig. 3:**
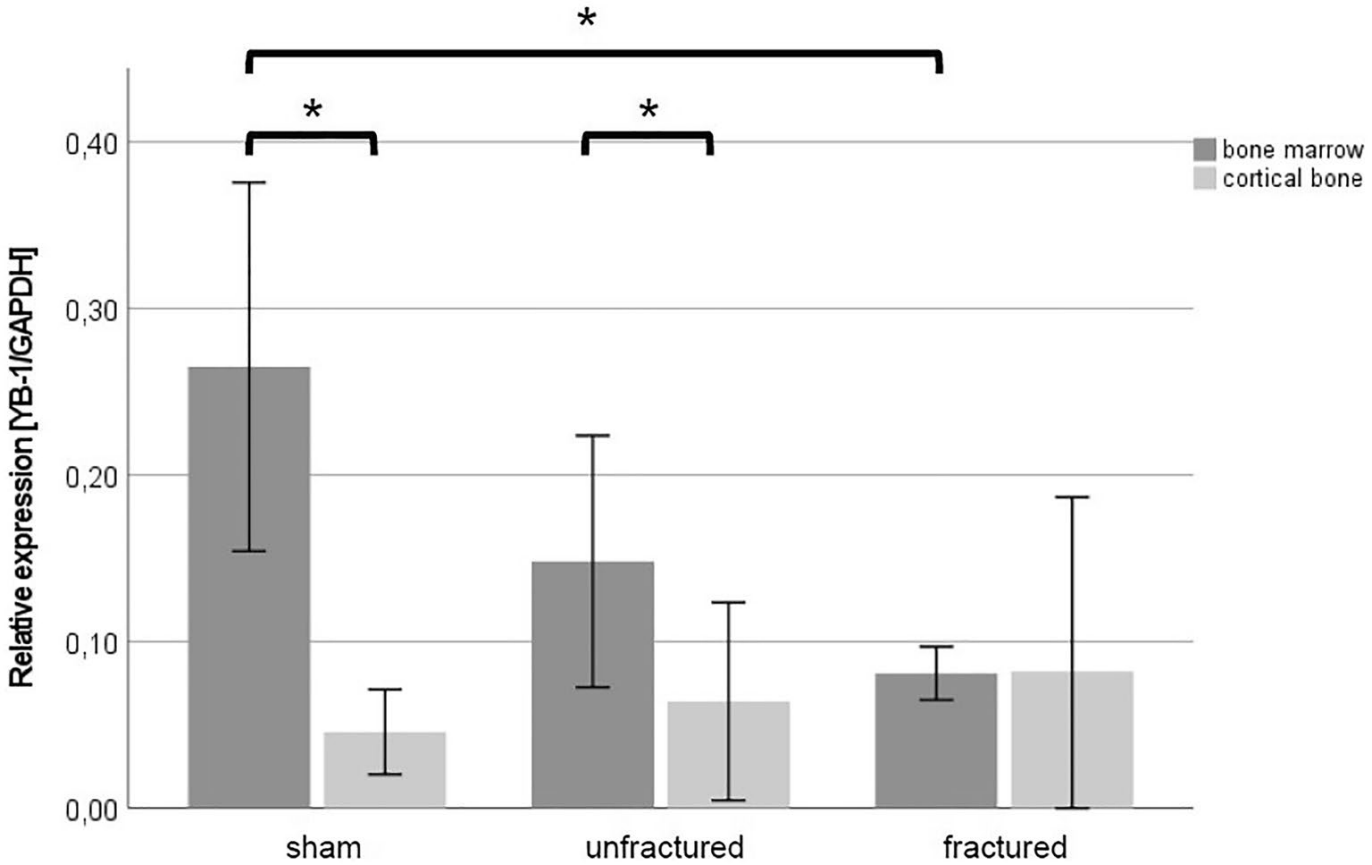
Relative YB-1 protein concentrations (*n* = 3, reference gene: GAPDH) presented with the mean and standard error of the mean. *p < 0.05

BM concentrations of YB-1 at the fracture site were significantly lower than those of the sham animals (MT + TH: 0.08 ± 0.1 vs. sham: 0.27 ± 0.1; *p* = 0.03). Interestingly, the BM concentrations of YB-1 in the tibias of the sham animals were higher than those in BM from both, the unfractured and fractured tibias of the MT + TH animals. These differences were not statistically significant. In contrast, there was no difference regarding YB-1 concentrations among the study groups in samples from the CB. When the YB-1 concentrations in BM and CB were compared, the sham animals had significantly higher YB-1 concentrations in BM than in CB (CB: 0.05 ± 0.02 vs. BM: 0.27 ± 0.1; *p* = 0.036). This was also the case with the unfractured tibias of the MT + TH animals (CB: 0.06 ± 0.05 vs. BM: 0.15 ± 0.07; *p* = 0.033). However, there was no statistically significant difference between YB-1 concentrations in CB and BM from the fracture site (*p* > 0.05; Fig. 3).

### Relative EpoR concentrations

**Fig. 4:**
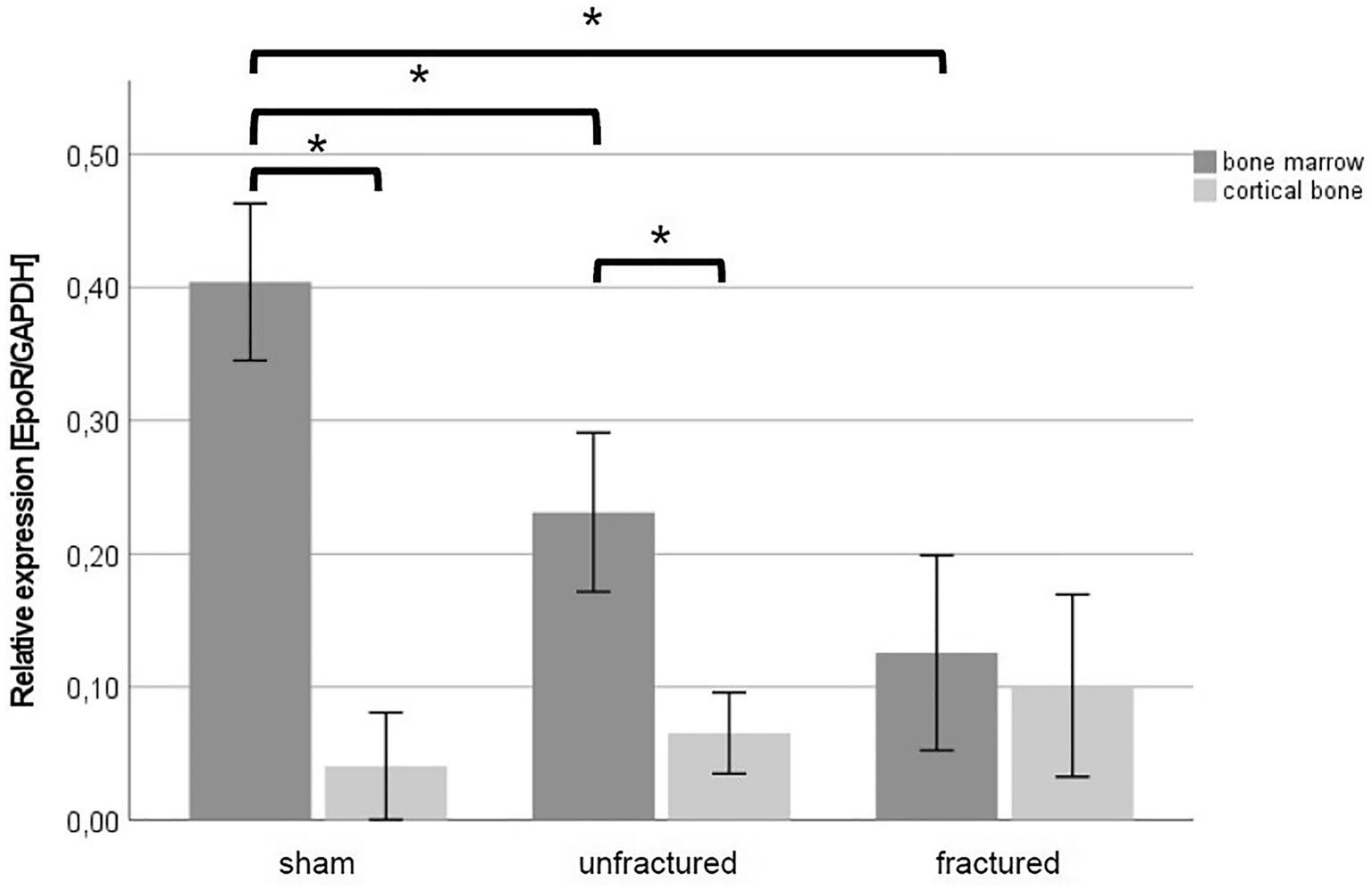
Relative EpoR concentrations (*n* = 3, reference gene: GAPDH) presented with the mean and standard error of the mean. *p < 0.05

BM concentrations of EpoR in the fracture zone were significantly lower than those in the sham group (MT + TH: 0.13 ± 0.06 vs. sham: 0.40 ± 0.05; *p* = 0.004). Furthermore, relative BM EpoR concentrations in unfractured bones were also lower as those in the sham group (MT + TH: 0.23 ± 0.05 vs. sham: 0.40 ± 0.05; *p* = 0.015). There was no significant difference between the study groups in CB concentrations of EpoR. BM EpoR concentrations were significantly higher than CB EpoR concentrations in the sham animals (CB: 0.04 ± 0.03 vs. BM: 0.40 ± 0.05; *p* = 0.015) and in unfractured bones in the MT + TH animals (CB: 0.07 ± 0.03 vs. BM: 0.23 ± 0.05; *p* = 0.026). There was no significant difference in BM and CB EpoR concentrations in fractured bones in the MT + TH group (*p* > 0.05; Fig. 4).

### Epo concentrations

**Fig. 5:**
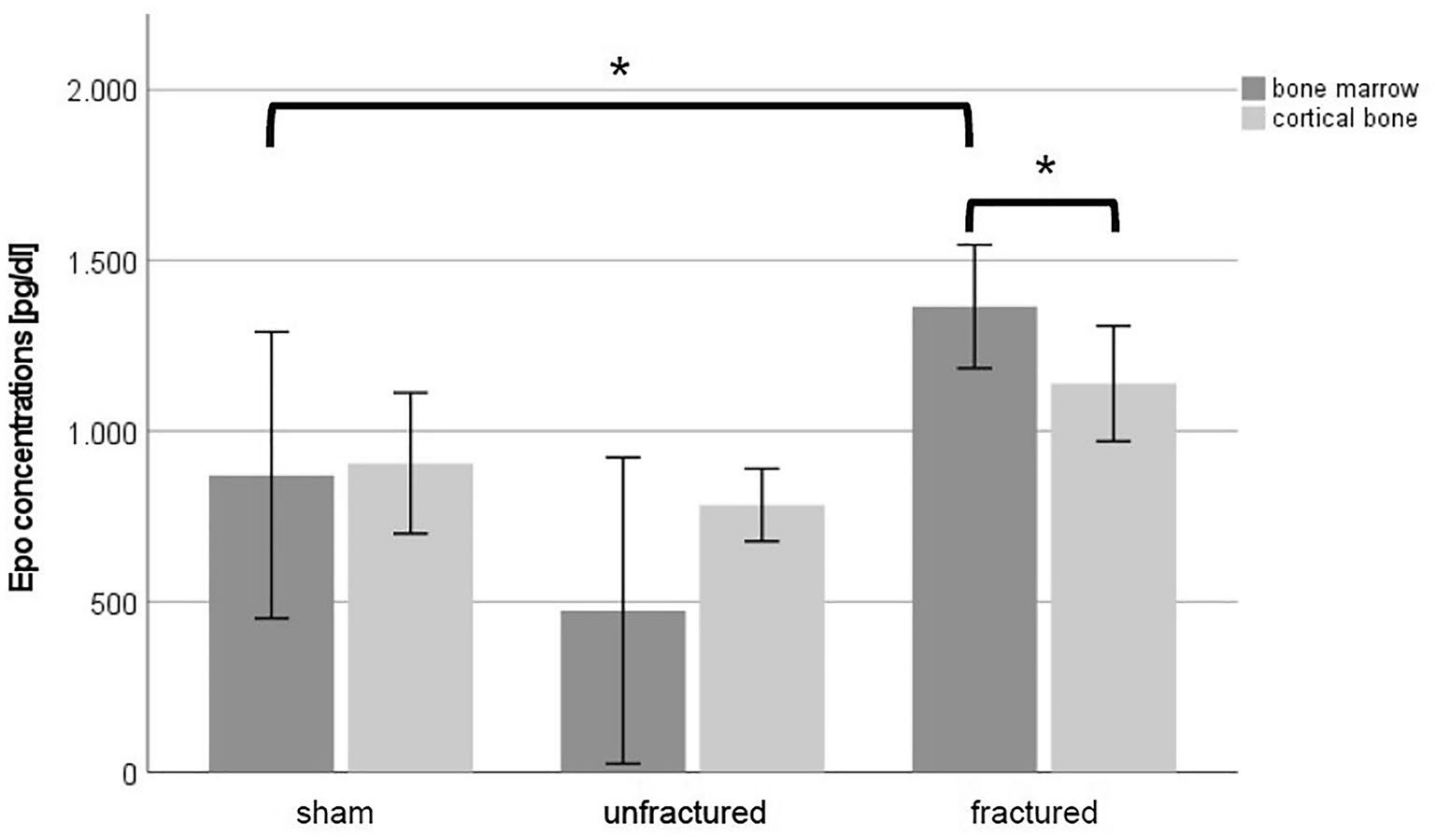
Absolute Epo concentrations (*n* = 8) presented with the mean and standard error of the mean. *p < 0.05

BM Epo concentrations in the fracture zone were significantly higher compared to those in the sham animals (MT + TH: 1438 pg/dL ± 237 vs. sham: 800 pg/dL ± 570; *p* = 0.025) (Fig. 5). There were no significant differences in CB Epo concentrations in the unfractured tibias in the sham group versus those in the MT + TH group (*p* > 0.05). BM Epo concentrations were significantly higher than CB Epo concentrations in the fracture zone of the MT + TH animals (CB: 1139 pg/dL ± 238 vs. BM: 1438 pg/dL ± 237; *p* = 0.022).

## Discussion

**Fig. 6:**
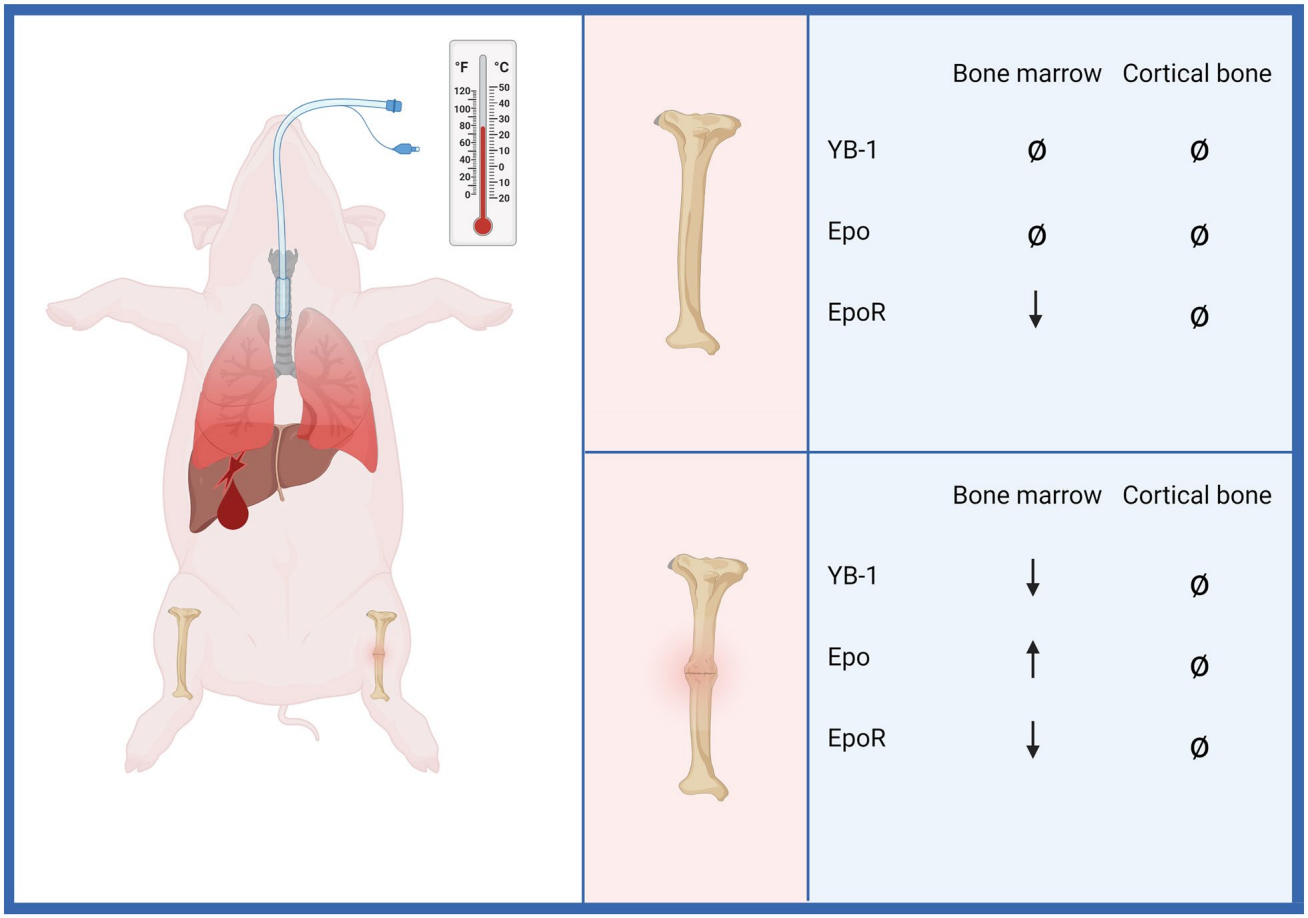
Illustration of the study results

Fractures are commonly associated with damage to both the surrounding soft tissues and blood vessels [3]. The impaired supply of oxygen and nutrients leads to local tissue hypoxia at the injury site [3]. At the same time, the formation of a fracture hematoma rich in signal proteins occurs. This hypoxic local microenvironment at the fracture site is characteristic of the onset of bone healing. Subsequently, immune cells, such as leukocytes and macrophages, migrate into the fracture gap and induce an inflammatory milieu, which triggers the formation of granulation tissue [18]. Clinical and experimental studies have provided evidence that local inflammation might be altered in cases of concomitant injuries and a systemic post-traumatic inflammatory response, thereby affecting the fracture healing process [6, 7, 19–21]. Therefore, characterization of early local healing processes under these conditions is of great interest [22]. The present study is the first study to investigate changes in the Epo-EpoR-YB-1 signaling axis within two bony compartments (BM and CB) in a clinically relevant large animal model of MT + TH. The findings are summarized as follows (Fig. 6):

- Reduced YB-1 concentrations in BM in the fracture zone of the MT + TH group, with accompanying higher Epo levels compared to those in the sham group, reflect local hypoxia.
- EpoR concentrations in BM of fractured and unfractured bones of the MT + TH group were lower than those in the sham group.
- Concentrations of Epo, EpoR, and YB-1 in CB were significantly lower than BM concentrations.

### Roles of YB-1 and Epo in fracture healing

Among hypoxia-related factors, YB-1, a member of the cold shock protein family and a regulator of chemokines involved in bone metabolism (e.g., RANTES/CCL5), has been identified as a novel binding factor for hypoxia-responsive elements [9, 23].

In a cell experiment under hypoxic conditions, Rauen et al. showed that YB-1 depletion led to an increase in Epo concentrations and, vice versa, higher YB-1 concentrations are accompanied by a reduction in Epo concentrations [9]. However, this regulatory function of YB-1 operated exclusively in the presence of hypoxia and not under physiological conditions [9]. A negative correlation between YB-1 and Epo concentrations was also shown in our data and is consistent with a hypoxia-induced change in protein concentrations in the early phase of fracture healing. This hypoxic condition might be most likely caused by ruptured vessels and TH-related reduced blood flow at the fracture zone.

YB-1 plays a role as an antagonist of hypoxia-inducible factor 1-alpha (HIF1α). The HIF1α transcription factor is upregulated by hypoxia and activates VEGF [24, 25]. This pathway plays a major role in the initiation and regulation of angiogenesis and the migration of mesenchymal stem cells during the early phase of fracture healing [26]. Several in vivo and in vitro experiments have shown that hypoxia-induced HIF1α activation leads to bone morphogenetic protein 2 (BMP-2) upregulation in osteoblasts [27–29]. Furthermore, members of the HIFα family are the major inducers of Epo transcription and are therefore responsible for increasing systemic Epo concentrations; however, the remote influence of HIF1α and HIF2α on Epo expression exhibits tissue specificity and age dependence [30, 31]. While in fetuses and neonates, the liver as well as the kidney are Epo producers, a rapid shift is observed after birth toward the peritubular cells of the kidneys as major producers [32]. However, in mouse models, it was shown that local production of Epo in osteoblasts as well as macrophages is also important for physiological bone and hematopoietic metabolism [33–36]. In addition, VEGF-induced upregulation of Epo production has been extensively described in the literature as the other major regulatory mechanism. Lower YB-1 concentrations in the early phase of fracture healing in the MT + TH group compared to those in the sham group in our study reflect local hypoxia in the fracture zone and represent an essential mechanism for the initialization of fracture healing. However, detailed studies on the hypoxia transcriptome must be evaluated according to individual injury patterns and the severity of the injuries. Previous research demonstrated significant alterations of local protein concentrations depending on the trauma load and the injury pattern [7]. We did not investigate the HIFα signaling pathways and their remote effects, as this was outside the remit of the present study. Nevertheless, our observations could be explained by both the activation of the HIFα-Epo signaling pathways and/or by upregulation of Epo gene expression locally as well as in the peritubular cells of the kidneys due to the lack/reduction of binding of YB-1 to regulatory HREs [31].

### The Epo-EpoR axis

The effect of Epo is mediated partially by the EpoR, which is expressed in proliferating, prehypertrophic, and hypertrophic zones of developing growth plates as well as in cartilaginous callus of healing bone [37]. The functional EpoR receptor is expressed in endothelial cells, osteoclasts, osteoblasts, and BM stem cells. Little is known about the function of endogenous Epo in osteoblasts. However, in a transgenic murine model with specific EpoR deletion in osteoblasts, Suresh et al. demonstrated that Epo signaling via the EpoR is an important regulatory mechanism in bone hemostasis and that osteoblast differentiation is directly controlled by this pathway [34]. In contrast, several animal studies demonstrated that high doses of exogenous Epo treatment reduced the bone mass of long bones by direct activation of osteoclasts. In this context, the effect of exogenous Epo seems to be dependent on the dose and the extent of bone injury [38]. The importance of the Epo-EpoR signaling pathway in terms of bone healing was also demonstrated by Wan et al., who observed a significant decrease in cartilaginous callus formation in a murine EpoR knockout model, providing strong evidence for an effect of Epo on chondrocytes [37]. Furthermore, biosynthesis of proteoglycan, accompanied by upregulation of chondrogenic marker genes, including SOX9, SOX5, SOX6, collagen type 2, and aggrecan, was inhibited by knockdown of the EpoR [37]. Therefore, EpoR might contribute to delayed healing or even nonunion of fractured bone. The family of SOX transcription factors, particularly SOXD:SOX5/6 and SOXE:SOX8/9 groups, is essential for chondrocyte differentiation and chondrogenesis, with the genes encoding these proteins expressed throughout chondrogenesis [39].

There is increasing evidence of a functional interaction between osteoblasts and key regulators, such as RUNX2, STAT3, and OSX/SP7 [40]. Walrafen et al. demonstrated a feedback mechanism between Epo and the EpoR in an in vitro experiment on human leukemic cells (UT-7), with binding of Epo and the EpoR leading to subsequent internalization and degradation of the EpoR [41]. Kumar et al. proved significant downregulation of the EpoR expression on mononuclear cells in a small cohort of patients (*n* = 19) who had sustained MT + TH [42, 43]. The authors suggested possible post-traumatic failure of the hematopoietic system due to an excessive proinflammatory cytokine milieu and elevated levels of circulating catecholamines altering the behavior of the BM microenvironment of the MT + TH patients [42, 43]. These findings are in line with those of a previous study by our group and demonstrate the translational relevance of the MT + TH model [7].

Systemic inflammation and the hypoxic stimulus of traumatic hemorrhage may also lead to chronically elevated systemic Epo concentrations, which simultaneously increase osteoblast as well as osteoclast activity and may lead to a reduction in bone mass [44]. In addition, we detected lower EpoR expression and simultaneously higher Epo concentrations in the fracture zone of the MT + TH animals compared to the sham group. On one hand, this could reflect an intact feedback mechanism in the MT + TH group as reported by Walrafen et al. On the other hand, lower Epo concentrations were observed in the nonfractured bones in the MT + TH group compared with the sham group [41]. A comparable trend, albeit without evidence of statistical significance, was also demonstrated with respect to the relative YB-1 concentration. However, these observations could also be explained by the impact of the injury pattern on bone metabolism and local vascularization in the animal model used. However, due to the absence of different trauma/injury groups (fracture, thoracic trauma and hemorrhagic shock), it remains unclear whether one of the individual injuries or the systemic immune response after MT is responsible for the changes in the unfractured bones. Based on our observations, further studies on the influence of specific injuries, injury patterns, as well as the severity of these injuries on the microenvironment and cell activation in fractured bones are urgently needed. Thus, this could provide more information on the role of the Epo–EpoR axis in terms of timely fracture healing. Alterations of the Epo–EpoR axis (especially in osteoprogenitor cells, granulocytes, and osteoblasts) due to a systemic inflammatory response after severe trauma could be a cause for impaired fracture healing.

### CB versus BM

Due to the different regenerative capacities of CB and BM [45, 46], we focused on both YB-1 and Epo concentrations in this study. In the study, although BM YB-1 concentrations in fractured bone in the MT + TH group were lower than those in the sham group, a similar finding was not detected in CB YB-1 concentrations. Furthermore, BM Epo concentrations in fractured bone were significantly higher than Epo concentrations in CB of the same side and in the BM in the sham group. Due to the superior vascularization in BM and higher metabolic rate of this compartment, BM might react very sensitively to hypoxic conditions [47]. Additionally, the higher Epo concentrations might be resulting of a combined accumulation of systemic Epo as well as an increased local production of resident cells after the hypoxic stimulus of the traumatic hemorrhage. In contrast to the cortex, which is nourished by numerous small vessels that ramify through the cortex (Haversian canals), the marrow cavity is filled with spongy bone that has about 10 times the surface area of compact bone [48]. Thus, the BM cavity affords a range of vascular niches that are thought to regulate the growth and differentiation of hematopoietic and stromal cells [48]. Against this background, the altered EpoR concentrations in BM versus the constant EpoR concentrations in less vascularized CB seem logical.

## Limitations

Several limitations of the present study need to be addressed. First, due to the duration of the observation period, we could only study the early phase of fracture healing. In addition, we used juvenile pigs, which limits the transferability of the results to adult animals and humans. Unfortunately, cell–cell communication and the underlying intracellular mechanisms could not be investigated and should be addressed in future studies. Furthermore, the standard deviations and standard errors indicate that larger sample sizes will be necessary for future animal experiments with this focus. Moreover, results could differ for different injury severities and for different injury patterns [7]. Another limitation of our work is that the origin (endogenous vs. kidney-derived) of the Epo studied could not be distinguished. Overall, the experimental setup reliably reproduces the clinical situation of a severely injured patient and allows for the first time the alterations of Epo, EpoR, and YB-1 concentrations within the hypoxia transcriptome under clinically realistic conditions in multiple trauma.

## Conclusions

To our knowledge, this is the first study that provides evidence that MT + TH alters BM and CB concentrations of YB-1, Epo, and EpoR in both fractured and unfractured bones. It can be assumed that changes in the local hypoxia transcriptome in the fracture zone can lead to a disturbance of fracture healing due to the systemic reaction after MT + TH. It remains unclear which individual injuries or injury patterns are responsible for these changes. The specific effects of both the fracture itself and the concomitant injuries on the hypoxia transcriptome need further investigation. In this regard, the roles of both the Epo–EpoR axis and hypoxia-responsive elements should be further elucidated. Moreover, future studies should focus not only on the resident cell population of both compartments (BM and CB) but also on the cellular crosstalk between them.

## Data Availability

The data and experimental protocols are available from the corresponding author upon request.

## Abbreviations

BM: Bone marrow
CB: Cortical bone
Epo: Erythropoietin
EpoR: Erythropoietin receptor
HIF: Hypoxia-inducible factor
HRE: Hypoxia-responsive element
MT: Multiple trauma
TH: Traumatic hemorrhage
VEGF: Vascular endothelial growth factor
YB-1: Y-box binding protein 1
